# Linking Self-Control, Hope, Positivity Ratio, Anxiety and Handwashing Habits during the Coronavirus Outbreak

**DOI:** 10.3390/ijerph19148859

**Published:** 2022-07-21

**Authors:** Shira Bukchin-Peles, Tammie Ronen

**Affiliations:** 1 Department of Agricultural & Resource Economics, University of California, Berkeley, CA 94720-3310, USA; 2 Department of Geography, The Hebrew University of Jerusalem, Jerusalem 91905, Israel; 3 The Glocal Program in International Development, The Hebrew University of Jerusalem, Jerusalem 91905, Israel; 4 Department of Social Work, Tel-Aviv University, Tel-Aviv 69978, Israel; tamie@tauex.tau.ac.il

**Keywords:** COVID-19, self-control, hope, anxiety, positivity ratio, hand washing

## Abstract

The novel COVID-19 is an infectious disease caused by the coronavirus. In the early stages of the pandemic, Israel struggled to contain its local outbreak through various measures that have virtually halted daily life and placed tens of thousands of people into quarantine. This study explored the role played by self-control and hope for obtaining two desired outcomes: (1) maintaining one’s positivity ratio (having more positive than negative affect—an indicator of wellbeing) and (2) increasing one’s contagion-preventing behavior (in this study—handwashing habits). Path analysis was conducted using survey data collected from 537 Israeli adults. Our findings suggest that self-control’s association with the positivity ratio is mediated through hope and anxiety levels. Self-control positively correlates with hope levels and negatively correlates with anxiety levels. Higher hope levels correlate with a higher positivity ratio, while lower anxiety levels correlate with a higher positivity ratio. The relationship between self-control and handwashing habits was mediated by hope, anxiety, and existing handwashing habits. This study brings research a step forward in demonstrating the vital role of positive components in achieving desired psychological and behavioral outcomes during an anxiety-provoking epidemic outbreak. In addition to its theoretical innovation, the importance of this study lies in its practical value: We focus on variables that are influenced by policy, education, and communication.

## 1. Introduction

During the winter of 2019–2020, a highly contagious novel coronavirus labeled COVID-19 started spreading rapidly worldwide, without any specific vaccines or medical treatments available to halt contagion (World Health Organization—WHO, 2020). In response to the local coronavirus outbreak in Israel, the government gradually imposed increasingly strict restrictions in a range of domains holding vast implications for citizens’ daily routines. At the time of the current data collection in mid-March 2020, these entailed: recommendations to the public to refrain from international travel; a 14-day mandatory quarantine for anyone returning from abroad or exposed to a confirmed coronavirus case; closed schools and businesses; bans on public gatherings; guidelines to cease unessential work; and extensive limitations on outings outside the home [1]. These restrictions virtually halted daily life and placed tens of thousands of Israelis into quarantine. Alongside these measures, media announcements were disseminated to educate citizens on how to protect themselves and others from infection, thereby preventing and slowing down community transmission of the virus. Israelis were instructed to frequently wash their hands or use an alcohol-based rub, practice respiratory etiquette when coughing or sneezing, refrain from touching the face, avoid close contact with people through social distancing, stay home, and self-isolate [2].

In such times of uncertainty and concrete personal risk, people’s rates of anxiety, stress, and fear of the unknown usually increase, especially during disruptions to familiar routines [3,4,5]. New studies also showed psychological distress in different forms amongst people exposed to the COVID-19 pandemic [6,7,8]. The current study focused on two desired outcomes during this period of anxiety and risk: (1) the role of self-control in maintaining one’s positivity ratio (having more positive than negative affects) and (2) the role of self-control in increasing prevention behavior (handwashing habits). Therefore, the positivity ratio and handwashing habits are the outcomes of this study.

To better understand how to achieve these outcomes, we chose to investigate the role of two positive components: hope and self-control. Some anxiety may be needed to prompt rapid behavioral changes in the short term (i.e., the recommended health-protective handwashing habits); however, excessive anxiety may decrease wellbeing, which can affect resilience in the long term. Positive components may also lead to behavior change and mediate anxiety, thus maintaining or even increasing wellbeing. The following section reviews the variables in our mediation model, examining the role of personal resources in promoting population resilience and behavioral change during the epidemic outbreak.

### 1.1. Positivity Ratio

In the current study, we use the concept of positivity ratio to denote wellbeing. The positivity ratio conceptualization derives from the idea that positive emotions and negative emotions operate as independent bipolar constructs so that the existence of one does not necessarily point to a lack of the other [9,10].

Positive emotions include pleasant or desirable situational responses, ranging from interest and contentment to love and joy. Positive affect is highly associated with wellbeing [11,12]. It is considered a marker of overall wellbeing or happiness, but positive emotions also enhance future growth and success [13]. The experiencing of positive emotions is associated with better functioning and, in the long run, correlates with enhanced physical, intellectual, and social resources [14]. Positive emotions are, therefore, a crucial component for achieving resilience. Several popular measures are used to quantify positive emotions (e.g., [10,15,16]).

Theoretical grounds for the importance of investigating positive emotions take root in Fredrickson’s [17] broaden-and-build theory, which describes positive emotions as “opening people up” and making them more resilient, creative, and open to changes and new experiences [18]. Positive emotions build enduring bio-psychosocial resources that support the ability to recover better from adverse situations. Additionally, by broadening one’s repertoire of more adaptive thoughts and actions, a crucial by-product is positive emotions’ undoing effect, which acts as an antidote that can undo or reduce the influence of negative emotions [19].

Positive affect correlates with satisfaction in life, high self-confidence, and a more prosperous social life [20]. Negative affect (e.g., including anger, hostility, stress) correlates with reports of stress symptoms [20,21]. Negative emotions promote survival at moments of threat by generating specific life-preserving actions (i.e., fight or flight). However, positive emotions promote survival in the long run by building resources for coping with life’s inevitable adversities [22].

Everyone has both negative and positive emotions. So, the question is not whether people have positive emotions but how high their ratio of positive to negative emotions is. Positive and negative affect are often studied as part of a positive–negative scale [10]. A higher ratio of positive to negative emotions has been identified as an index of human wellbeing and even flourishing [23]. Previous studies linked positive emotion in general and a high positivity ratio in particular to reductions in fear and anxiety [24] as well as to self-control [25,26].

### 1.2. Self-Control

Self-control is regarded in this study as an essential personal skill influencing hope, anxiety, handwashing habits, and positivity ratio. The current model draws on Rosenbaum’s [27] conceptualization of self-control as a learned repertoire of goal-directed skills that enable humans to act upon their aims, postpone gratification, and overcome difficulties relating to thoughts, emotions, and behaviors. Self-control allows individuals to consciously decide to take charge of their behavior [28]. This process is prompted by situations where automatic and habitual responses are interrupted or ineffective.

Rosenbaum [29] described four categories of self-control behaviors: (1) use of cognitions and “self-statements” to control emotional and physiological responses; (2) application of problem-solving strategies such as planning, problem definition, alternative evaluation, and anticipation of consequences; (3) ability to delay immediate gratification; and (4) perceived self-efficacy. Accordingly, he maintained that the first function of self-control is redressive and is directed at controlling responses such as anxiety or pain. This function is essential for coping with stress.

Self-control skills have been linked not only to reductions in stress but also to the ability to attain wellbeing, happiness, and positive emotions [24,30]. Therefore, self-control skills are of great importance to human psychological health as they involve a crucial personal component of coping with stressful events as well as a major element for becoming resilient [31,32,33]. Self-control necessitates that people assess disruptions in their habitual way of thinking, believe that their actions can improve their coping, and expect themselves to be capable of creating the desired change [27,29,34]. Thus, self-control skills are crucial for both personal (i.e., emotional) and interpersonal (i.e., social) flourishing [30].

Based on previous studies linking self-control to lower rates of anxiety [24,35,36,37], we hypothesized that self-control would be directly linked with lower anxiety related to the coronavirus outbreak. During recent decades, self-control has been linked not only to reductions in anxiety but also to increases in wellbeing in general [24,38,39] and increased hope in particular [40]. Thus, we expected that self-control skills would be linked with the level of hope and with a higher positivity ratio, despite the stressful situation.

Rosenbaum [28] maintained that the second function of self-control is reformative. Accordingly, it facilitates the adoption of new types of behaviors that require delaying gratification, resisting temptations, and developing new habits. Thus, we also hypothesized that self-control would be linked with better handwashing habits regularly.

Note that self-control is a learned repertoire or skill set that is needed when obstacles need to be overcome, when one faces difficulties, when new behaviors need to be learned, or when habitual response sequences are interrupted or proven ineffective [27,34,41,42]. Self-control is therefore related to the original handwashing habits one had before the pandemic. If people regularly washed their hands, it would be easier to keep doing so during the pandemic. We hypothesize that self-control would relate to habitual behavior, which, in turn, mediates its relation to the contagion-preventing behavior.

### 1.3. Hope

Broadly, scholars define hope as something positive that one wishes would happen in the future [43]. However, hope is a complex and comprehensive concept. We follow Snyder’s [44] definition of hope as “the perceived capability to derive pathways to desired goals and motivate oneself via agency thinking to use those pathways”. Thus, hope involves agency, which is one’s belief in bringing about change and targeting a specific goal, and it also involves pathways—different ways to achieve that goal. Snyder outlined hope as a dynamic cognitive, motivational system and, thus, as more than just a positive emotion. Hope encompasses a concept that helps human beings to look toward a better future; enhances coping with difficulties, sickness, trauma, and disaster; and serves as a critical component in attaining wellbeing [45,46,47,48].

In the current study, we hypothesize that hope influences the positivity ratio and handwashing habits during the epidemic. Several theoretical arguments support these hypotheses. First, hope has been significantly linked with taking action and problem solving [44]. Hope is specifically needed to take action in situations where one believes the chances of success are limited [49]. Studies over the past decade have supported the importance of hope among adults, demonstrating that high hope serves as a buffer against stress and trauma (see Snyder et al. [50]).

Valle et al. [51] presented hope as a psychological strength for human coping. Hope was found to mediate psychological distress’ associations not only with health status but also with life satisfaction [52,53]. Hope was also shown to mediate the negative potential of fear [54]. In health psychology, studies revealed that people with high levels of hope exhibit more constructive thinking about how to address their problems [55]. Studies suggest that the level of personal hope spurs anticipation of a better future; thus, more hopeful people would be encouraged to set more complicated and ambitious goals and take action [44,46,49]. Accordingly, during an epidemic outbreak, when people are facing uncertainty, fear, and actual risk, people who are more hopeful may attempt to take more action in order to increase the chances of staying safe. We suggest they are likely to wash their hands in more situations even if they are not sure about the odds of success. Given their ability for constructive thinking and for overcoming negative emotions such as fear, people with higher levels of hope may also reveal a higher positivity ratio (i.e., experiencing more positive than negative emotions).

### 1.4. Epidemic-Related Anxiety

Human beings are regularly exposed to change, crisis, and anxiety. While some traumatic experiences seem to increase mental health problems, it appears that other traumatic experiences, paradoxically, seem to affect them positively [3]. Two main trends have characterized research studying anxiety, stress, and coping. The environmental approach conceptualizes changes and stress as essential factors influencing the formation of psychological disorders [56]. The second group of studies, in line with a resilience approach, asserts that people respond “normally” even to severe crises. These studies suggest that although anxiety may manifest as an increase in the frequency of behavior problems, usually people do not develop post-traumatic stress disorder after exposure to trauma or stress. They typically return to their usual patterns of behavior, relating to the event as a challenge [37,57]. Exposure to the substantial stressors accompanying the coronavirus outbreak may cause an increase in anxiety. We hypothesize that anxiety will be linked to a reduction in positive emotion. Nevertheless, we hypothesize that anxiety will also be linked to desired prevention behavior—in this case, to the increase in contagion-preventing handwashing habits.

### 1.5. Mediation Model

We predicted several mediation paths based on the hypotheses thus far. First, we hypothesized that hope would mediate self-control’s links with both the positivity ratio and handwashing habits during the outbreak. We also expected that baseline pre-outbreak handwashing behavior (pre-HW) would mediate the link between self-control and mid-outbreak handwashing behavior (mid-HW). Lastly, we hypothesized that anxiety would mediate self-control’s links with both mid-HW and positivity ratio.

## 2. Method

### 2.1. Sample and Procedure

The survey was conducted over one week during the COVID-19 pandemic outbreak in Israel (16–22 March 2020) among 537 adult respondents, 50% women, ages 18–80 years (*SD* = 13.80), using a professional online panel service, Panel4All (http://www.panel4all.co.il/ (accessed on 2 April 2020)). The Panel4All service recruited a representative sample of the adult Israeli population, considering sex, age, and residential area.

The Tel Aviv University Institutional Review Board gave its ethical approval for the study. All the participants expressed their informed consent to participate in the study after being guaranteed anonymity.

Regarding the stage in the epidemic outbreak during data collection, when the online survey period began (16 March), Israel had reported 298 confirmed cases of COVID-19, 4 of whom were in severe condition, but no deaths yet, while the global death toll moved past 7000. By the end of the sampling period (22 March), the reported number of confirmed cases had risen to 1071, with 18 persons in severe condition and 1 death. At the time of the survey, schools, public buildings, and most businesses had been shut down. Government directives had been issued instructing citizens to leave their homes only to shop for food and medication, but enforcement was not yet strict. Throughout the sampling period, only 30% of the workers were defined as essential and permitted to go to work. Unemployment rose dramatically from 3.5% to 16.0% over the survey week. Immediately before the survey, many Israelis had reluctantly canceled their previous week’s Purim holiday travel plans, and the traditional festive public celebrations had been banned. Most Israelis were complying with the governmental directives, remaining in their homes.

### 2.2. Measures

#### 2.2.1. Hope

We used Snyder and Sympson [48] cognitive, goal-oriented measure, the Hope Scale (also known as the Goals Scale). The scale was translated and validated by Dubrov [58]. This scale includes four agency items (e.g., “My past experiences have prepared me well for my future”), four pathways items (e.g., “I can think of many ways to get out of a jam”), and four fillers (distractors) that are not included in the total hope score but are intended to make the scale content less obvious. Prior studies revealed the scale’s sound internal reliability, with Cronbach alphas ranging from 0.74 to 0.88. Its convergent validity has been substantiated by its predicted correlations with several other scales designed to measure similar concepts [59]. In the present study, the Cronbach alpha was 0.89.

#### 2.2.2. Self-Control Skills

The 36-item adult version of the Self-Control Scale [29] was designed to assess self-control skills, including problem-solving skills, attentional control/distraction, cognitive reframing, delay of gratification, and use of self-talk and self-reinforcement. Participants rated items (e.g., “When I am faced with a difficult problem, I try to approach it systematically”) on a scale ranging from −3 (not characteristic of me at all) to +3 (very characteristic of me). The score is derived by summing the answers (while subtracting reverse-coded items that are negatively worded). Thus, higher scores indicate higher self-control skills. Note that the scale was initially developed in Israel [29] and, therefore, is validated for the Israeli population. In the present study, the Cronbach alpha was 0.84.

#### 2.2.3. Positivity Ratio

The 20-item Positive and Negative Affect Schedule [10] is a self-reporting checklist of affect adjectives designed to provide independent measures of positive and negative affect, with ten items each. The scale was translated and validated by Ben-Zur [60]. Participants rated the frequency at which they experienced the 20 emotions in the last week (e.g., positive: excited, proud; negative: frustrated, sad) on a scale ranging from 1 (never) to 5 (always). In the current study, internal consistency was α = 0.86 for positive emotions and α = 0.84 for negative emotions. To compute the students’ positivity ratio, the mean score for the positive affect subscale was divided by the mean score for the negative affect subscale. A larger ratio (higher score) represents a greater number of positive over negative emotions.

#### 2.2.4. Handwashing Habits before (Pre-HW) and during (Mid-HW) the Outbreak

A handwashing checklist was presented twice to participants, once regarding handwashing habits at the current time during the epidemic outbreak (mid-HW) and once retrospectively regarding their routine handwashing behavior before the outbreak (pre-HW). We asked participants to mark all circumstances in which they currently/previously washed their hands on the following list: before every meal; after every meal; during food preparation; before and after wound treatment; after blowing my nose, coughing, or sneezing; after touching trash; after using the toilet; during my travels; after meeting people in public places; when I arrive home; after any contact with an animal or with animal feces; after changing diapers or cleaning a child who has used the toilet. Participants were asked to mark all applicable answers. For the analysis, we summed up the number of marked answers specified.

#### 2.2.5. Epidemic-Related Anxiety Level

To assess stress reactions to the pandemic, anxiety was coded using qualitative content analysis of participants’ responses to an open-ended question: “What is your feeling and mood regarding the coronavirus epidemic outbreak?”. Two research assistants separately examined the answers and classified them into five levels ranging from Very calm and positive (1) to Very anxious (5), mainly by counting the use of keywords. Next, the two raters discussed cases of disagreement. The inter-rater agreement was very high: they agreed on the tendency of the relation (i.e., calm anxious or neutral) in 94% of the statements and gave the exact same score in 86% of the cases.

#### 2.2.6. Socio-Demographic Background

Socio-demographic data were also collected by asking participants for their age, sex, years of formal education, household size, and residential area.

### 2.3. Data Analysis

After examining pre-HW versus mid-HW habits using *t*-tests for paired samples, path analysis was conducted to test the hypothesized mediation model using IBM SPSS Amos. The model’s fit to the data was evaluated using the criteria of χ^2^/df ≤ 3, a comparative fit index (CFI) ≥ 0.95, Tucker–Lewis coefficient (TLI) ≥ 0.95, and a root mean square error of approximation (RMSEA) < 0.08 [61]. The bootstrap method was utilized to test for indirect effects (i.e., mediation), with the confidence level set at 0.95 and bootstrap bias-corrected samples set at 5000. When zero is not in the 95% confidence interval (CI), the indirect effect is significantly different from zero at two-tailed *p* < 0.05 [62]. For each specific mediation effect, we calculated a separate *z*-score, as suggested by Taylor, MacKinnon, and Tein [63]. Suppose the *z*-score is between −1.96 and +1.96. In that case, the null hypothesis of no mediation cannot be rejected, whereas if the *z*-score falls outside that range, it is possible to reject the null hypothesis, namely, indicating the existence of an indirect effect. Lastly, we calculated each mediation path’s specific standardized estimates and significance using the “Indirect Effects” AMOS Plugin [62]. The plugin uses 2000 bootstrap samples and 95% confidence intervals.

## 3. Results

Table 1 displays the study variables’ means, standard deviations, and maximum and minimum values.

### 3.1. HW Habits

As seen in Table 1, the paired-samples *t*-test conducted to compare pre-HW habits (*M* = 5.41, *SD* = 2.80) vs. mid-HW habits (*M* = 6.76, *SD* = 2.72) yielded a significant difference, *t*(536) = 14.33, *p* < 0.01 (2-tailed). This finding suggested that the coronavirus outbreak did affect HW habits by significantly increasing the number of circumstances (e.g., when returning home, after eating, after meeting people, rather than only after using the bathroom) in which people washed their hands. Thus, pre-HW was included in the subsequent path analysis.

### 3.2. Path Analysis

**Figure 1:**
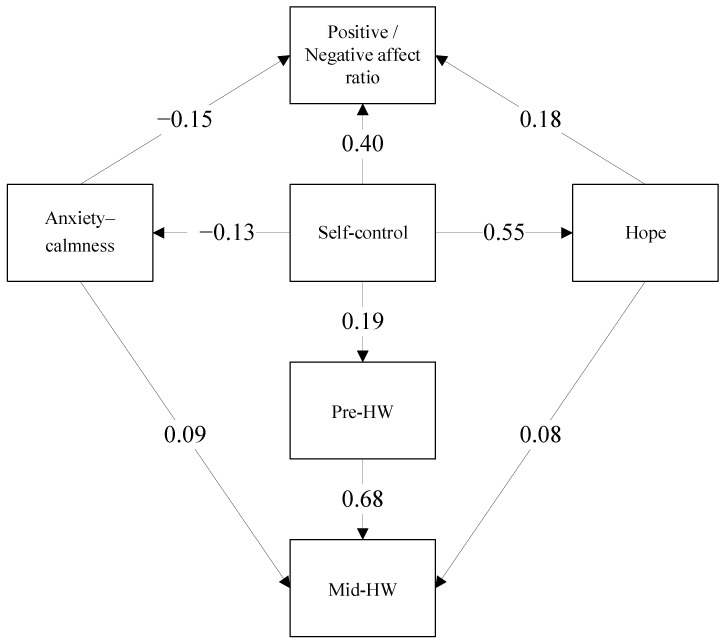
The mediation model linking self-control skills to personal resources, epidemic-related anxiety, and handwashing (HW) habits. Standardized regression weights are presented. All paths are significant.

Path analysis indicated that the theorized model depicted in Figure 1 provided a good fit to the data on the following fit indices: χ^2^/df = 0.95 (χ^2^ = 5.72, *p* = 0.455), CFI = 1.00, TLI = 1.00, RMSEA = 0.00. Table 2 displays the estimated regression weights.

The complete mediation model is displayed in Figure 1. The mediated effect of self-control on the positivity ratio was 0.12. This is in addition to the direct effect as seen on the figure (B = 0.40, SE = 0.04, *p* < 0.001). Bias-corrected bootstrap analysis of indirect effects indicated a significant indirect association between self-control and positivity ratio (*p* < 0.001, two-tailed, SE = 0.024). The boundaries of bootstrap confidence intervals for this indirect effect were 0.078 and 0.172. Further analysis confirmed both double-path mediation chains, as the *z* scores fell outside the −1.96 and +1.96 range, both for the path of self-control→hope→positivity ratio (*z* = 4.14) and for the path of self-control→anxiety→positivity ratio (*z* = 2.42). The indirect effects differed significantly from zero, and the null hypothesis of no mediation could be rejected.

We also calculated each mediation path’s specific standardized estimates and significance. For the path of self-control→hope→positivity ratio, the estimate was B = 0.102 (*p* = 0.001). The confidence intervals for this indirect effect were between 0.70 and 0.139. For the path of self-control→anxiety→positivity ratio, the estimate was B = 0.019 (*p* = 0.003). The confidence intervals for this indirect effect were between 0.007 and 0.041.

The correlation between self-control and pre-HW was significant and positive (B = 0.194, SE = 0.042, *p* < 0.001). The mediated effect of self-control on mid-HW was 0.16. Bias-corrected bootstrap analysis of indirect effects indicated a significant indirect association between self-control and mid-HW (*p* < 0.001, two-tailed, SE = 0.033). The boundaries of bootstrap confidence intervals for this indirect effect were 0.095 and 0.227.

Further analysis confirmed all three double-path mediation chains, as the *z* scores fell outside the −1.96 and +1.96 range: for the path of self-control→hope→mid-HW (*z* = 2.45), for the path of self-control→anxiety→mid-HW (*z* = −2.13), and for the path of self-control→pre-HW→mid-HW (*z* = −4.51). The indirect effects differed significantly from zero, and the null hypothesis of no mediation could be rejected.

We also calculated each mediation path’s specific standardized estimates and significance. For the path of self-control→hope→mid-HW, the estimate was B = 0.042 (*p* = 0.024). The confidence intervals for this indirect effect were between 0.012 and 0.071. For the path of self-control→pre-HW→mid-HW, the estimate was B = 0.131 (*p* = 0.001). The confidence intervals for this indirect effect were between 0.084 and 0.178. For the path of self-control→anxiety→mid-HW, the estimate was B= −0.012 (*p* = 0.004). The confidence intervals for this indirect effect were between −0.025 and 0.004. Note that this specific path is nonsignificant based on bootstrapped confidence intervals [64].

Thus, all the hypotheses were supported: Hope was found to mediate the link between self-control and both the positivity ratio and mid-HW. Pre-HW was found to mediate the link between self-control and mid-HW. Anxiety was found to mediate the link between self-control and both mid-HW and positivity ratio.

We hypothesized that the relation between self-control and mid-HW would be mediated through pre-HW, hope, and anxiety (see Introduction). Nevertheless, we also analyzed this relation. The link between self-control and mid-HW was found to be nonsignificant (B = 0.048, SE = 0.038, CR = 1.265, *p* = 0.206).

Note that we also examined age, gender, household size, and education level and the predictor (self-control). None of these variables were found to significantly affect self-control: education (B = 0.054, SE = 0.044, *p* = 0.221), household size (B = −0.024, SE = 0.043, *p* = 0.581), gender (B = 0.009, SE = 0.043, *p* = 0.839, note, men = 1), age (B = 0.085, SE = 0.044, *p* = 0.054). We were expecting a more significant result for the relation of age and self-control because self-control develops over the years. However, the sample is composed of only an adult population, which may explain the results.

## 4. Discussion and Conclusions

In line with a human resilience approach, our mediation model examined the role of personal resources in promoting population wellbeing and health-protective behavioral change during the COVID-19 epidemic outbreak. With regard to the desired changes in self-reported behavior, as expected, our findings suggested that in response to national and international health directives issued during the coronavirus outbreak, Israeli adults did significantly broaden the situations in which they regularly washed their hands, compared to the pre-outbreak period.

Overall, the current path analysis findings indicated the indirect effect of better self-control skills on people’s higher positivity ratio—their psychological wellbeing—as mediated through their stronger hope levels and lower anxiety. Similarly, the path analysis indicated the indirect effect of better self-control skills on people’s increase in contagion-preventing behavioral habits during the outbreak (mid-HW)—as mediated through stronger hope, lower anxiety levels, and higher pre-outbreak hygienic routines. This study brings research a step forward in demonstrating the important role of positive components—self-control and hope—in achieving the desired psychological and behavioral outcomes during an epidemic outbreak.

Self-control skills have been previously associated with routine desired behaviors such as washing hands (see, e.g., Baldeh [65]) and with increases in hope and positive emotions [25,30]. However, the current results highlighting self-control’s role in promoting desired mental and physical health indices expand on past research that usually focused on these skills’ primary role in decreasing various kinds of undesired behaviors and anxiety as well [24,30,36]. The current findings underscore the possible multifaceted role played by adults’ ability to follow orders, cope with demands, delay temptations and focus on hope and positive emotion despite the stressful situation. During the epidemic outbreak, it seemed that the public handled the situation, yet long-term examination is needed to test the long-term effects of this event.

Regarding psychological health, such self-control skills may not only help reduce people’s anxiety about the epidemic and its consequences but also may foster greater hope about their sense of agency and specific pathways toward a better future—both of which may promote people’s capacity to maintain a higher ratio of positive to negative emotions, to achieve better wellbeing. A higher positivity ratio may, for example, help people be creative (e.g., initiate online conversation) and overcome social distancing, which is strange specifically to the Israeli population. Moreover, regarding physical health, greater self-control skills’ impact in reducing anxiety and fostering hope (as well as in increasing people’s baseline everyday hygiene habits before the epidemic outbreak) may, at the same time, facilitate the desired changes in the public’s preventative handwashing behaviors. Self-control is needed in order to establish healthy handwashing habits, which are neither compulsive nor loose. Nevertheless, the path of self-control→anxiety→mid-HW was found to be significant according to the *z*-score calculations but nonsignificant based on bootstrapped confidence intervals. Some researchers argue that *z*-score calculations are less accurate as they assume a normal distribution for the indirect effect, leading to incorrect interpretations of significance testing of mediation [64].

The mediating role played by hope in promoting resilience and health during an outbreak may be attributed to hopeful people’s higher likelihood of taking action and solving problems [44,49,54,55]. Such individuals are therefore likely to anticipate that more handwashing during the outbreak would improve their lives despite the associated challenges, and they may consequently decide to adopt this behavior, hoping for a better future [44,46,49].

As expected, anxiety played a mediating role too. As linked to lower positivity ratio, the current finding substantiates prior research indicating that anxiety impairs human wellbeing and coping [3,4]. As linked to increases in contagion-prevention hygiene habits, the current finding coincides with research showing that people experiencing high anxiety try to find a solution for the root cause of their anxiousness. Further research should explore whether the degree of handwashing habits increases rationally during an epidemic outbreak, in order to prevent infection as recommended by the authorities, or if it became an irrational over-washing compulsion.

## 5. Study Limitations and Implications

Self-control, positive emotions, and hope have been associated with several positive outcomes related to health, success, and wellbeing (see reviews by Snyder [44], Honken, et al. [66], Neimiec [67], and Peterson and Seligman [68]). The findings of this study emphasize, once again, the importance of positive personal components to human coping even in times of global crises. In addition to its theoretical innovation, the importance of this study lies in its practical value: We focus on variables that are influenced through policy, education, and communication. Hence, policies, interventions, and cultural practices aimed at strengthening these personal resources are expected to boost the welfare of the population as a whole, as well as that of individuals [69]. Future research should continue to investigate the precise types of education and intervention that may encourage the desired outcomes.

The association between personal resources and management of an epidemic outbreak should be further explored to cover other types of epidemics in different communities and ages, as well as other positive components. Future studies should also rely on larger and more diverse samples and in different times of the outbreak (e.g., overtime at different stages of the lockdown or after returning partially to work), to validate and broaden the findings of the current study. The HW checklist should also be complemented with other measures. Notably, the sampling method used for this preliminary investigation—online survey—was an unavoidable mid-outbreak methodology considering the lockdown conditions; however, the generalization of the current findings may be limited because this sample represents a specific population who answers internet surveys. Self-reporting and post facto techniques are known to have weaknesses such as response biases, selective memory, positive self-attribution, and exaggeration of motivations [70]. Thus, future similar studies should be carried out in different countries and investigate different settings.

## Figures and Tables

**Table 1:** Means, standard deviations, and maximum and minimum values of study variables (*n* = 537).

	Self-Control	Hope	Positivity Ratio	Anxiety–Calmness	Handwashing	
					Before the Epidemic (Pre-HW)	During the Epidemic (Mid-HW)
*M*	22.06	47.03	1.54	3.90	5.41	6.76
*SD*	25.88	9.98	0.62	0.89	2.80	2.72
Min.	−66	8	0.53	1	0	0
Max.	90	64	4.80	5	11	12
Cronbach α	0.84	0.89	Positive: 0.82			
			Negative: 0.84			

**Table 2:** Estimated regression weights (*n* = 537).

Variable	Estimate	Standard Error	Critical Ratio	*p*
Self-control→Hope	0.551	0.036	15.297	<0.001
Self-control→Anxiety–calmness	−0.129	0.043	−3.013	0.003
Self-control→Pre-HW	0.194	0.042	4.581	<0.001
Self-control→Positivity ratio	0.398	0.043	9.250	<0.001
Pre-HW→Mid-HW	0.675	0.031	21.62	<0.001
Hope→Mid-HW	0.077	0.031	2.456	0.014
Hope→Positivity ratio	0.185	0.043	4.312	<0.001
Anxiety–calmness→Mid-HW	0.094	0.031	3.009	0.003
Anxiety–calmness→Positivity ratio	−0.148	0.036	−4.111	<0.001

## Data Availability

The data that support the findings of this study are available from the corresponding author, Shira Bukchin-Peles, upon reasonable request.
